# Triple-Band Square Split-Ring Resonator Metamaterial Absorber Design with High Effective Medium Ratio for 5G Sub-6 GHz Applications

**DOI:** 10.3390/nano13020222

**Published:** 2023-01-04

**Authors:** Mohammad Lutful Hakim, Mohammad Tariqul Islam, Touhidul Alam, Sharul Kamal Abdul Rahim, Badariah Bais, Md. Shabiul Islam, Mohamed S. Soliman

**Affiliations:** 1 Pusat Sains Ankasa (ANGKASA), Institut Perubahan Iklim, Universiti Kebangsaan Malaysia (UKM), Bangi 43600, Selangor, Malaysia; 2 Department of Electrical, Electronic and Systems Engineering, Faculty of Engineering and Built Environment, Universiti Kebangsaan Malaysia (UKM), Bangi 43600, Selangor, Malaysia; 3 Department of Computer Science and Engineering (CSE), International Islamic University Chittagong (IIUC), Kumira, Chattogram 4318, Bangladesh; 4 Wireless Communication Centre, Universiti Teknologi Malaysia, Skudai 81310, Johor, Malaysia; 5 Faculty of Engineering (FOE), Multimedia University, Persiaran Multimedia, Cyberjaya 63100, Selangor, Malaysia; 6 Department of Electrical Engineering, College of Engineering, Taif University, P.O. Box 11099, Taif 21944, Saudi Arabia; 7 Department of Electrical Engineering, Faculty of Energy Engineering, Aswan University, Aswan 81528, Egypt

**Keywords:** metamaterial absorber, square splits ring resonator, 5G sub-6 GHz applications, effective medium ratio

## Abstract

This article proposes a square split-ring resonator (SSRR) metamaterial absorber (MMA) for sub-6 GHz application. The unit cell of the MMA was designed and fabricated on commercially available low-cost FR-4 substrate material with a dielectric constant o 4.3. The higher effective medium ratio (EMR) of the designed unit cell shows the compactness of the MMA. The dimension of the unit cell is 9.5 × 9.5 × 1.6 mm^3^, which consists of two split rings and two arms with outer SSRR. The proposed MMA operates at 2.5 GHz, 4.9 GHz, and 6 GHz frequency bands with a 90% absorption peak and shows a single negative metamaterial property. The E-field, H-field, and surface current are also explored in support of absorption analysis. Moreover, the equivalent circuit model of the proposed MMA is modelled and simulated to validate the resonance behavior of the MMA structure. Finally, the proposed MMA can be used for the specific frequency bands of 5G applications such as signal absorption, crowdsensing, SAR reduction, etc.

## 1. Introduction

The metamaterial is a sub-wavelength artificial material that exhibits unusual electromagnetic (EM) behavior, such as negative permittivity or permeability and negative or positive refractive index [1]. These properties make metamaterial-based microwave devices extremely popular for various applications such as energy harvesters [2], filters [3], sensors [4,5,6,7], polarization converters [8], invisible clocks [9], antenna design [10,11,12,13,14,15], SAR reduction [16], absorber [17], and photonic devices [18,19,20,21]. Metamaterial also significantly enables 5G wireless communication, which will be widely used for producing various 5G devices [22]. Currently, 5G communication is developing rapidly beyond expectation. Lower frequency bands are widely used in LTE/4G; the higher-frequency mm-wave frequency band is still under experimental exploration. Upcoming 5G communication will be implemented in sub-6 GHz or 5G mid-band frequency [23]. For 5G (fifth-generation) communication, 2.5/2.6 (B41/N41) GHz, 3.7–3.98 GHz, 4.94–4.99 GHz license, and 5.9–7.1 GHz unlicensed frequencies are allotted from the sub- 6 GHz band by the federal communication commission (FCC). The MIIT of China officially announced the 2.5/2.6 (B41/N41) GHz 3.3–3.6 GHz and 4.8–5 GHz frequency bands. The maximum bandwidth requirement for operating at this frequency is 40–100 MHz [23,24,25]. Therefore, there is a need to design a metamaterial absorber (MMA) to operate precisely at this frequency. However, most researchers have developed absorbers that either operate in an ultra-width band or show a random absorption peak [26]. This random absorption will change the device’s efficiency. In [24,27], thick multi-layer substrate MMAs for ultra-width absorption are presented, which operate at a 3.2–11 GHz and 2.2–5.83 GHz frequency, respectively. A broadband sectional resonator base MMA is presented in [28] for a 7.18–8.8 GHz frequency. In [29], a mandarin line base broadband MMA presented for 1.84–5.96 GHz. The authors of [29] present an inkjet-printed PET substrate-based broadband MMA (1.0–4.5 GHz). An origami-based microwave absorber is presented in [30] for reconfigurable absorption bandwidth from 3.4 to 18 GHz frequency. Besides the triple absorption band, MMAs are offered in [31,32,33,34,35] with various patch designs. Most MMAs used FR-4 substrate, and complex patch designs also suffer from larger sizes (electrical wavelength). Several types of metallic ring configurations have been used to achieve triple absorption bands, such as two rings (3.36 GHz, 3.95 GHz, and 10.48 GHz) [31]; three concentric metallic resonators (3.95 GHz, 5.92 GHz, 9.21 GHz) [32]; six distinct concentric rings (1.75 GHz, 2.17 GHz, 2.6 GHz) [33]; triple circular slot ring (2.9 GHz, 4.18 GHz, 9.25 GHz) [34]; circular ring; and inner Jerusalem cross (4.4 GHz, 6.05 GHz, 13.9 GHz) [35]. Moreover, dual-band MMAs are presented in [36,37] where the overall bandwidth is very low and does not cover a sub-6 GHz unlicensed spectrum. In [24,27,31,32,33,34,35,36,37,38], the MMA’s EMR value is in the range of 2–9, where a higher EMR value is significant for designing a more compact structure for MMA.

Despite all these MMAs, there is a need for a new MMA design that will cover a sub-6 GHz license and unlicensed frequency spectra and can be used in upcoming 5G wireless communications. This research takes the initiative for designing such types of MMAs. This paper proposes triple-band MMAs, where the MMA can operate at 2.5 GHz, 4.9 GHz, and 6 GHz frequency bands with a narrow high absorption bandwidth.

## 2. Unit Cell Design and Analysis

**Figure 1:**
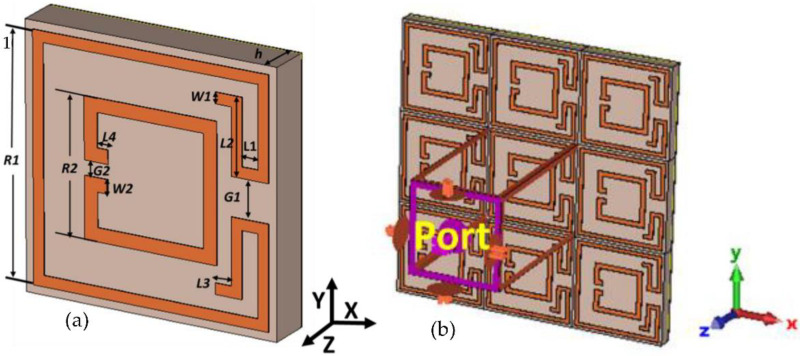
(**a**) Perspective view and (**b**) Simulation setup of proposed MMA.

**Figure 2:**
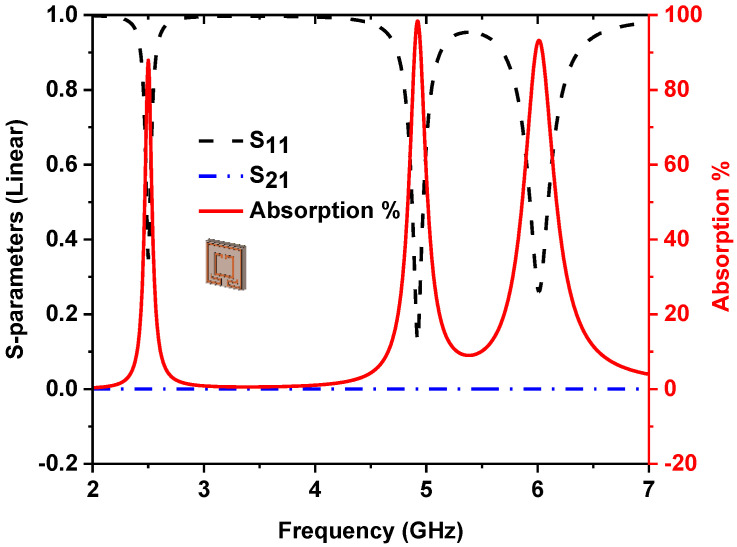
S-parameters and absorption property of the proposed MMA.

Figure 1 displays the proposed three-layer (metal-dielectric-metal) MMA. The copper has been used to design the MMA patch and ground layer. On the other hand, an FR4 substrate material with a dielectric constant of 4.3 and loss tangent of 0.002 was employed as dielectric substrate material. The proposed unit cell patch adjusts two complementary square rings with an additional adjacent arm. Simulation was accomplished utilizing commercially available computer simulation technology (CST) 2022 microwave studio software [39]. The default surface-based tetrahedral meshing was chosen to design the MMA, and the unit cell boundary conditions were applied in the x- and y-directions, and the transverse electric (TE) mode electromagnetic wave (EM) was applied towards the negative z-direction. The design parameters are tabulated in Table 1. The absorption property (*A*) of the projected MMA is determined by Equation (1) [40,41].

(1) $$A=1-S_{11}^{2}-S_{21}^{2}$$

where *S*_11_ and *S*_21_ are the transmission and reflection coefficients, respectively. The conductivity of the copper ground is σ = 5.8 × 10^7^ S/m resistivity *ρ* = 1.72 Ω-m and permeability *µ* = 1. The skin depth of the EM wave is estimated by $\delta =\sqrt{\rho /\pi f\mu }=0.0148\mathrm{mm}$. Therefore, the EM wave will be blocked by the 0.035 mm thick ground layer, and the transmission coefficient (*S*_21_) will be zero. Therefore, the absorption equation is

(2) $$A=1-S_{11}^{2}$$

where absorption (*A*) depends on the designed MMA’s reflection coefficient (*S*_11_). Figure 2 shows the designed MMA’s absorption and S-parameters curve.

**Figure 3:**
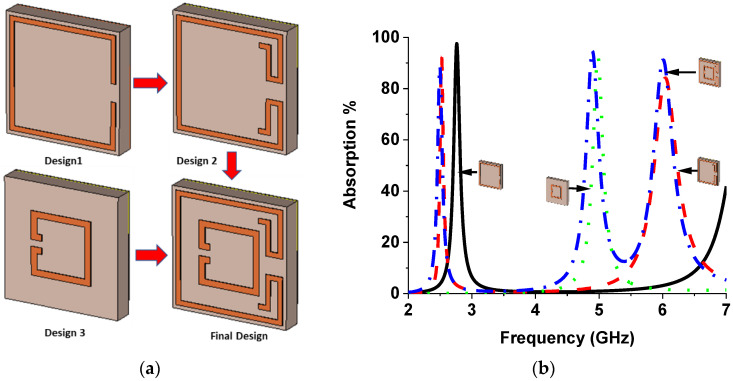
(**a**) Design evaluation of projected MMA, (**b**) absorption plot of design evaluation.

Evaluation of the unit cell resonator is revealed in Figure 3a to realize the adsorption behaviors of the projected MMA. The absorption curve of the various design steps is presented in Figure 3b, and the peaks and maximum absorption of different designs are listed in Table 2. A single square split ring resonator found a single absorption peak at 2.76 GHz resonant frequency. After adding additional adjacent parts at outer ring splits in design 2, two peak absorptions are found at the s2.52 and 6.04 GHz frequencies. A small square splits ring is used in design 3, which shows a single absorption peak at 4.98 GHz. The final design is prepared by combining design 2 and design 3, which offer three absorption peaks at 2.5, 4.9, and 6 GHz.

## 3. MMA Design Analysis

**Figure 4:**
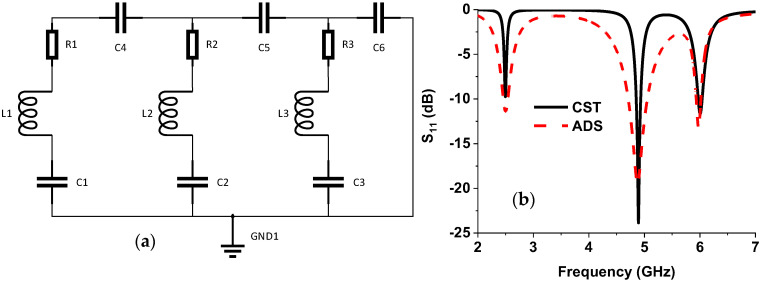
(**a**) Equivalent circuit diagram of the proposed MMA, L1 = 27 nH, L2 = 13.62 nH, 19.5 nH, C1 = 0.15 pF, C2 = 0.078 pF, C3 = 0.054 pF, C4 = 40.57 pF, C5 = 0.06 pF, C6 = 0.05 pF, R1 = 28.5 ohm, R2 = 40.5 ohm, and R3 = 23.5-ohm (**b**) S11 curve from CST and ADS.

An equivalent circuit of the projected absorber is described in Figure 4a [42,43,44], which was simulated by the Path-Wave Advanced Design System (ADS) software by Keysight [45]. The outer ring, the additional part attached to it, and the inner split ring represent an RLC circuit parallelly connected with the coupling capacitance between them. The inductance L1, L2, and L3 were calculated using Equation (3) from the outer ring, outer ring additional parts, and inner rings, respectively. In Equation (3), *L_s_* is the inductance, the length of the strip-line is *l*, the width of the strip-line is *W*, and *D* is the substrate thickness.

(3) $$L_{s}=0.00508l\left[\ln\left(\frac{2l}{W+D}\right)+0.5+0.2235\left(\frac{W+D}{l}\right)\right]$$

The associated capacitance C1, C2, and C3 are calculated by Equation (4) for lower, middle, and upper frequencies, respectively, where f is the resonance frequency.

(4) $$C_{s}=\frac{1}{4\pi^{2}f^{2}Ls}$$

Coupling capacitances C4, C5, and C6 are calculated by Equation (5), where conducting strip area is *A*, the distance between the two strips is *d*, and $\varepsilon_{r}$ $\varepsilon_{0}$ are the relative permittivity and free space permittivity.

(5) $$C=\varepsilon_{0}\varepsilon_{r}\frac{A}{d}$$

The associated resistance in the RLC circuit is determined by tuning for increment and decrement of the *S*_11_ value. The calculated values were also slightly adjusted to achieve a similar *S*_11_ curve to CST. Figure 4b shows the *S*_11_ parameter value of CST and ADS simulation.

## 4. Results Analysis

**Figure 5:**
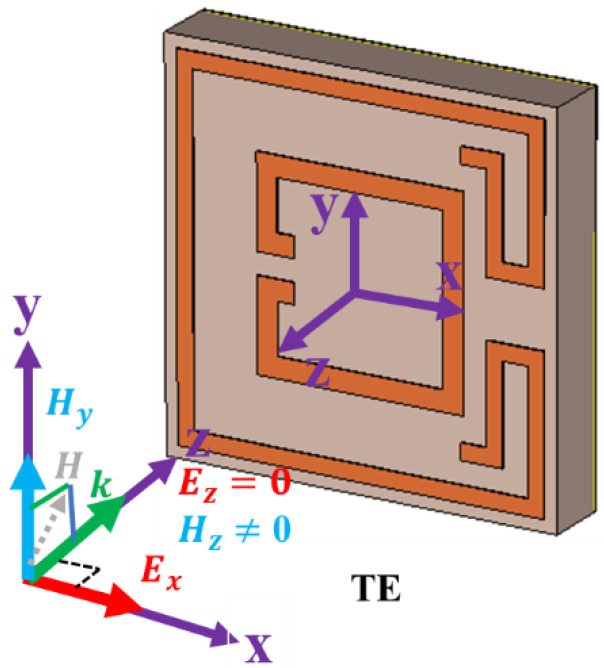
H-field and E-field vector directions for TE-mode incident EM wave.

The proposed MMA was simulated for the transverse electric (TE) mode of the EM wave. The H-field and E-field directions of the TE mode are presented in Figure 5, where $\vec{H}$ and $\vec{E}$ represent the H-field and E-field vector directions. There is no electric-field vector at the TE mode in the wave propagation direction ($\vec{k}$).

The reflection coefficient (*S*_11_) relay on significantly on the metamaterial’s effective impedance (*Z_Eff_*), presented in Equation (6), where *Z*_o_ is the free space impedance.

(6) $$S_{11}(\omega )=\frac{Z_{Eff}-Z_{0}}{Z_{Eff}-Z_{0}}$$

Effective impedance is as follows:

(7) $$Z_{Eff}(\omega )=\sqrt{\mu_{0}\mu_{r}(\omega )/\varepsilon_{0}\varepsilon_{r}(\omega )}$$

In Equation (7), *µ*_o_ and *ε*_0_ are the free space permeability and permittivity, respectively. The absorption property is also calculated by Equation (8) [46].

(8) A(ω)=4Re(Z)[1+Re(Z)]2+[Im(Z)]2

In Equation (8), the unity absorption will be accomplished for the state, Real|Z|≈1 and Imaginary|Z|≈0 because no reflection will happen. The normalized Impedance of the MMA is calculated by Equation (9) [47], which is characterized by frequency-dependent relative permeability and permittivity.

Normalized impedance is as follows

(9) $$Z=Z_{Eff}(\omega )/Z_{0}=\sqrt{\mu_{r}(\omega )/\varepsilon_{r}(\omega )}$$

Hence, impedance matching depends on the metamaterial property. The reflection coefficient (*S*_11_) and transmission coefficient (*S*_21_) are extracted from the CST simulation. The Nicolson–Ross–Wier method is used for calculating permeability (Equation (10)), and permittivity (Equation (11)), where wave number is $k_{o}=2\pi f/c$, the velocity of light is *c*, the thickness of substrate material is *d*, and *f* is the frequency. Permeability and permittivity are used to calculate the refractive index using Equation (12) [47,48,49].

Permeability:

(10) $$\varepsilon_{r}=\frac{2}{jk_{o}d}\left[\frac{\left(1-S_{11}-S_{21}\right)}{\left(1+S_{11}+S_{21}\right)}\right]$$

Permittivity:

(11) $$\mu_{r}=\frac{2}{jk_{o}d}\left[\frac{\left(1-S_{21}+S_{11}\right)}{\left(1+S_{21}-S_{11}\right)}\right]$$

Refractive index:

(12) $$n_{r}=\sqrt{\mu_{r}\times \varepsilon_{r}}=\frac{c}{j\pi fd}\times \sqrt{\frac{{\left(S_{21}-1\right)}^{2}-S_{11}^{2}}{{\left(S_{21}+1\right)}^{2}-S_{11}^{2}}}$$

**Figure 6:**
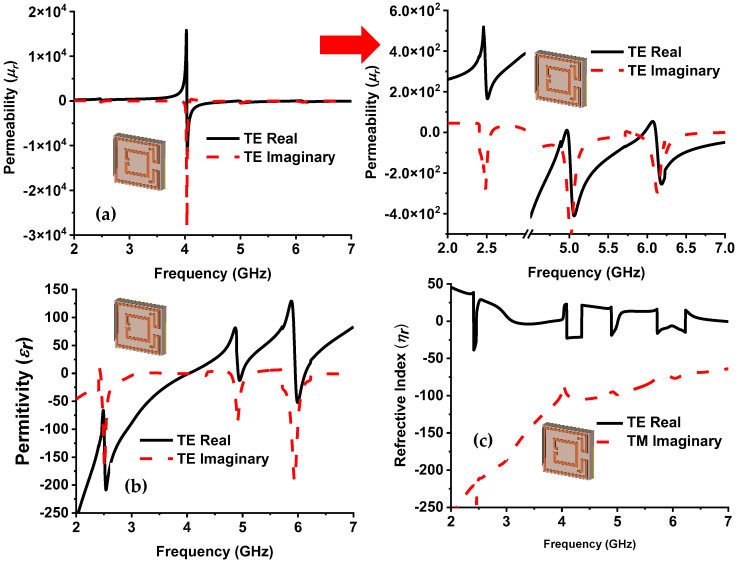
Metamaterial property of proposed absorber in the TE mode, (**a**) permeability, (**b**) permittivity, and (**c**) refractive index plots.

The proposed design’s transmission coefficient (*S*_21_) is zero, resulting in Equation (11); it is easily assumed that the negative permittivity is entirely dependent on *S*_11_ because *d* and *k_o_* are constant values. The square ring, splits, and gap settle the *S*_11_ of the proposed structure; therefore, all these parameters influence the capacitance and inductance of the resonator and alter the *S*_11_ value, which leads to negative permittivity. Figure 6a,b present the permittivity and permeability plot for TE modes, and the range of negative values (Real part) for both modes are listed in Table 3. At lower operating frequencies (2.47–2.52 GHz), the value of permeability is positive, but the permittivity is negative. Therefore, the lower frequency band has a single-negative (SNG) metamaterial behavior. The middle band (4.82–4.97 GHz) has negative permeability (4.82–4.95 GHz), and negative permittivity (4.925–4.99 GHz), from 4.925 to 4.95 GHz, has a double-negative (DNG) value. The upper band (5.9–6.11 GHz) showed permeability or permittivity negative, alternatively. Figure 6a,b show that the imaginary part of permeability and permittivity is negative at the operating frequency band. Both the real and imaginary values of permittivity and permeability are simultaneously important for the impedance matching of MMA. The refractive index is also calculated by Equation (12), which is shown in Figure 6c.

**Figure 7:**
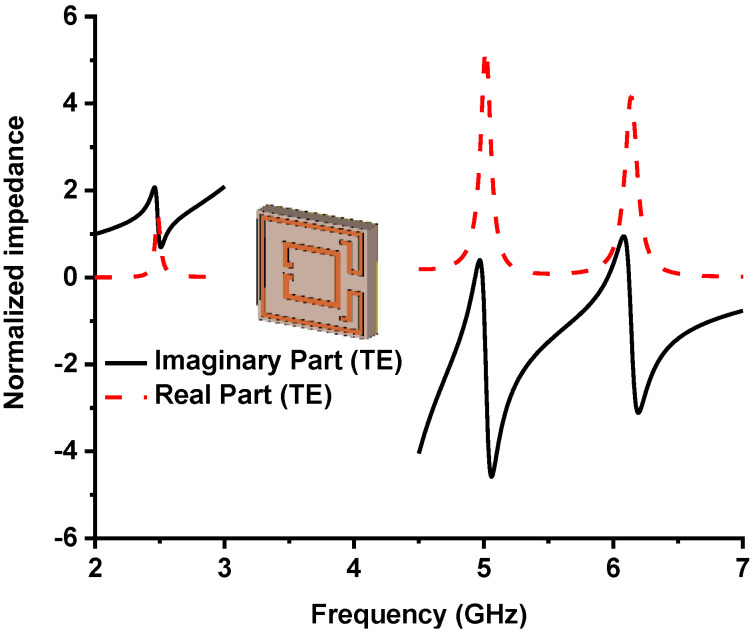
The normalized impedance of the proposed metamaterial absorber.

**Figure 8:**
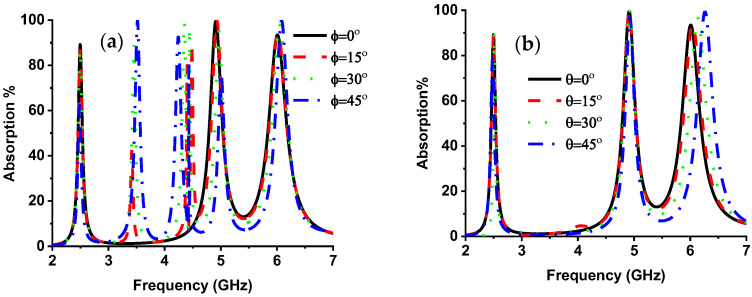
(**a**) Polarization incident angle (TE Mode) and (**b**) oblique incident angle (TE mode) of the proposed absorber.

The metamaterial property at the resonant frequency is shown in Table 4, where the real value of permeability and permittivity became alternatively negative at the resonance frequency. The complex value of permittivity and permeability achieved a negative refractive index at 4.9 and 6 GHz frequency. Figure 7 illustrates the normalized impedance plot. At 2.5, 4.9, and 6 GHz, resonance frequency. The imaginary, and real values of normalized impedance are near zero and unity, respectively, obtaining near-unity absorption at resonance peaks. The quality (*Q*) factor of the designed MMA is considered by $Q=f_{c}/\Delta f$, where fc is the center frequency, and Δ*f* is the full width at half maximum (FWHM). The designed MMA shows a *Q* factor of 62.5, 44.54, and 33.33 at 2.5, 4.9, and 6 GHz resonance frequencies, respectively, where corresponding FWHM are 40, 110, and 180 MHz. The *EMR* is an essential factor in compact metamaterial absorber design. The higher *EMR* value represents the compactness of MMA. The *EMR* of the designed MMA is 15, which is determined by Equation (13) and shows the very compact structure of the intended MMA [50]. The polarization angle investigation of the MMA is presented in Figure 8a, which provides unique absorption up to 15°; with an increment of polarization angle, the peak absorption is reduced in the middle absorption band [40,51]. The absorption plot for various oblique incident angles up to 45° is plotted in Figure 8b. The lower and middle bands show an absorption and upper-frequency peaks shifted towards higher frequencies. This happens due to the asymmetric structure of the MMA. The variation in polarization incident angle creates different electric and magnetic field intensities on the MMA patch, which causes frequency shifting.

(13) $$EMR=\frac{\mathrm{Wavelength}\;(\mathrm{mm})}{\mathrm{Lenght}\;\mathrm{of}\;\mathrm{the}\;\mathrm{Unit}\;\mathrm{cell}\;(\mathrm{mm})}$$

**Figure 9:**
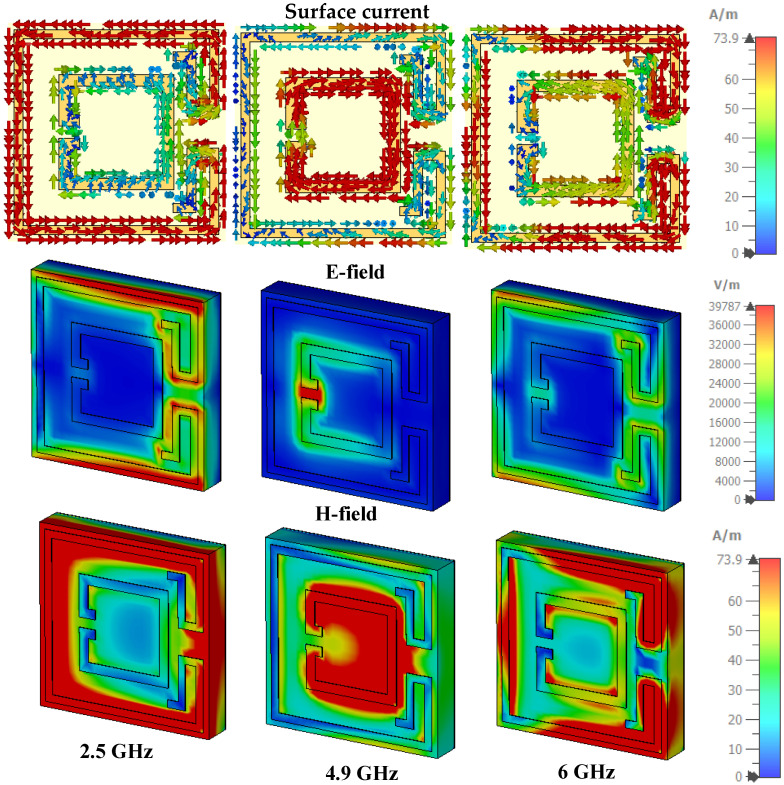
E-field, H-field Abs (absolute value) component, and surface current distribution (TE).

Absorption behavior can also be understood from a detailed discussion of the magnetic field, electric field, and surface current distribution. Figure 9 reveals the TE mode’s surface current allocation for three resonance frequencies. At 2.5 GHz resonant frequency, the current moves in the anticlockwise direction in the outer ring, where an additional bend portion injunction makes an anti-parallel flow. On the other hand, there are two types of current distribution in the inner ring. The upper, lower, and left arms (outer side) have a clockwise current flow, whereas the upper, lower, and right arms (inner side) have an anticlockwise tendency. Overall, this current flow makes an anti-parallel flow, which defines permeability as the cause of the magnetic resonance. The current distribution on the top layer expresses permittivity, which stands for the electrical part of resonance at 2.5 GHz resonant frequency. Figure 9 shows the surface current distribution of 4.9 GHz resonant frequency, where the current in the inner ring is rotating in a clockwise direction. In the outer ring, the left, upper, and lower arms (inner side) have an anticlockwise current flow; on the other side, the right, upper and lower arms (outer side) have a clockwise rotation. The adjacent part of the outer ring has an anti-parallel current flow with the inner and outer ring (right arm). The overall current flow makes an anti-parallel rotation, which generates a magnetic part of resonance at a 4.9 GHz resonant frequency. At 6 GHz resonance frequency, the current in the upper, lower, and right arms of the outer ring is rotating anticlockwise and in the clockwise direction in the left arm. The current in the inner ring is flowing anticlockwise. However, the current flow in the left arm of the inner ring is clockwise; at 6 GHz resonant frequency, the overall current flow is anti-parallel, as demonstrated in Figure 9.

The relation between current allocation, magnetic field and electric field in time-varying EM waves can be analyzed from Maxwell Equations (14) and (15) [52,53], where Equation (14) is Faraday’s law of EM induction. Equation (15) represents the modified form of Ampere’s law ∂D/∂t (displacement current).

(14) ∇×E=−∂B∂t

(15) ∇×H=J+∂D∂t

The relation of *E* and *H* vectors can be understood from Equations (16) and (17),

(16) D(t)=ε(t)×E(t)

(17) B(t)=μ(t)×H(t)

where B = magnetic flux density, D = electric flux density, and μ are the permittivity and permeability, respectively. Considering time dependence $e^{-j\omega t}$ and by placing time derivative jω in Equations (18) and (19), Maxwell’s equation is rewritten as,

(18) ∇×E=−jωμH

(19) ∇×H=jωεE

Figure 9 shows the e-field and h-field of TE mode at 2.5 GHz, 4.9 GHz, and 6 GHz. At a 2.5 GHz frequency, the e-field strength is higher in the upper and lower arm and adjacent parts of the outer ring. At a 4.9 GHz frequency, the e-field, concentered in the inner ring splits and left arm, is responsible for 99% absorption. The e-field is condensed in the outer ring left arm upper and lower corner, and the adjacent arm at 6 GHz frequency, which generates 97% absorption peaks. This type of solid e-field continues in contrast to the incident e-field, producing a stronger e-field than the incident e-field and producing electrical resonance [54]. Electric and magnetic resonance need to co-occur to get maximum absorption. Figure 9 presents the h-field distribution, where at 2.5 GHz resonant frequency, a strong h-field appears in the outer ring. At 4.9 GHz frequency, the h-field intensity is higher in the inner ring; on the other side, at 6 GHz resonant frequency, a strong h-field occurs in the right and left arm of the exterior ring. The e-field and h-field act together to achieve maximum absorption peak.

## 5. Measurement

**Figure 10:**
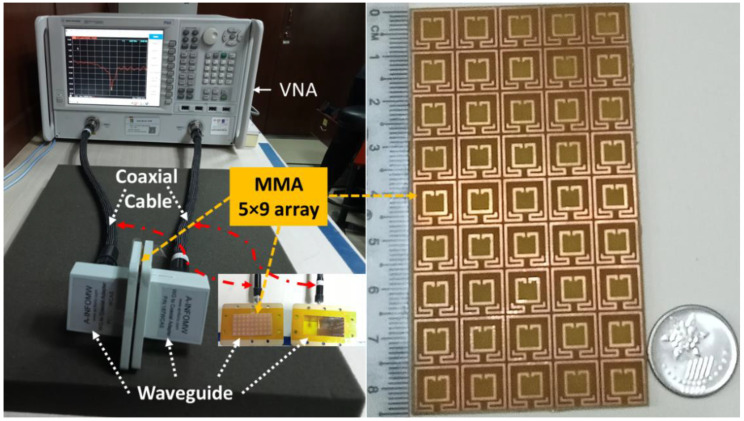
Measurement setup of the proposed MMA.

**Figure 11:**
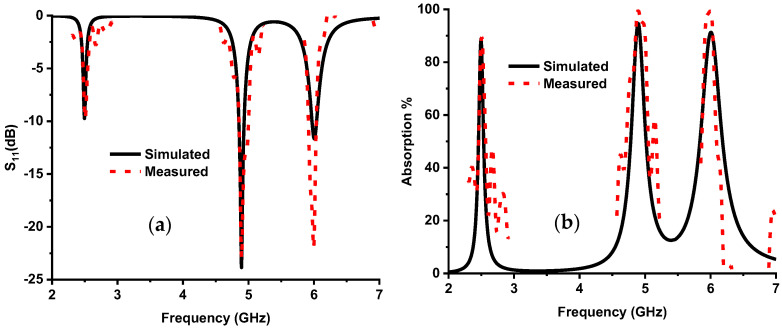
Simulated and measured results: (**a**) S11 parameters in dB, (**b**) absorption %.

The proposed absorber has been fabricated and measured. Figure 10 illustrates the measurement setup, where the Vector Network Analyzer (VNA) has been used for measurement. Three A-INFOMW WGs have been used for measuring each absorption band. The lower frequency band (2.5 GHz), the middle band (4.9 GHz), and the upper band (6 GHz) were measured using P/N:340WCAS, P/N:187WCAS, and P/N:137WCAS, respectively. The waveguide is connected to the VNA via a coaxial cable. Figure 11 shows the measurement result of S_11_ in dB and absorption as percentage, which is a reasonable adjustment with simulated data and validates the results of the designed absorber.

Table 5 shows an elaborate comparison with the present MMA, where the MMA’s patch design, size, substrate materials, operation frequency, absorption, and metamaterial property are recorded. The proposed MMA operates in three specific bands at a sub-6 GHz frequency, where References [27,38] have ultra-width absorption bands below 6 GHz. Ultra-width absorption bands are above 6 GHz in [24], which is not suitable for specific frequency applications. References [36,37] have dual-band absorption at sub-6 GHz frequency. Triple-band absorption was achieved in [31,32,34,35], but the upper band exceeds the sub-6 GHz frequency band. The metamaterial property has not been acknowledged except in [31], where the metamaterial attributes of the designed MMA are discussed in detail. The patch of the proposed MMA is less complex and smaller in size than other existing MMAs listed in Table 5, which makes the proposed one more cost-effective than others; furthermore, the one offered has an acceptable absorption peak compared with existing MMA.

The designed MMA can be applicable in the field of 5G sub-6 GHz antenna design, such as the mutual coupling reduction in MIMO antenna elements, by placing the MMA horizontally between two antennae [55,56] or vertically [57], RCS reduction, and EMI shielding [49], SAR reduction [58,59], IoT applications [60], microwave range sensing [61] etc. The existing sub 6-GHz MMA in [55,56] achieved a single frequency at 5.5 GHz and 5.1 GHz frequency with EMR values of 6.05 and 12, respectively. The fractal-based MMA in [57] shows absorption peaks at a 3.5 GHz frequency with an EMR of 9.96. Additionally, the proposed MMA achieved a higher EMR value than [49,58,59,61] and multiple absorption frequency bands, which make the proposed one preferable over existing MMAs in the range of sub-6 GHz frequency for 5G applications.

## 6. Conclusions

This paper presents a single-negative square split-ring resonator metamaterial absorber for the 5G sub-6 GHz license and the unlicensed frequency spectrum. The proposed MMA achieved 99% maximum absorption at 4.9 GHz frequency with high-quality factors at 2.5 GHz, 4.9 GHz, and 6 GHz resonance frequency. The EMR value of the designed MMA represents that the proposed one is more compact than the existing MMAs, which is vital for the size and cost of the device. The proposed MMA’s TE results and metamaterial property analysis are presented and discussed in detail. An equivalent circuit model is also presented, which will help designers in the upcoming generation of efficient absorbers for relative applications. Finally, a detailed comparison is also made, prioritizing the proposed one over existing work regarding size, absorption percentage, and specific frequency applications. Therefore, the proposed MMA can be utilized in mobile phones or other electronic devices to reduce SAR by absorbing EM waves and isolation reduction between two antennas. Additionally, it can be used in the energy-harvesting application of microwave frequency.

## Figures and Tables

**Table 1:** Parameter’s list of proposed MMA.

Parameters	Value (mm)
R1	8.90
R2	5.00
G1	1.40
G2	0.53
L1	0.60
L2	2.80
L3	0.60
L4	0.40
W1	0.40
W2	0.50
h	1.60

**Table 2:** Peaks and maximum absorption of different designs.

Design	Resonance Frequency (GHz)	Maximum Absorption Frequency (GHz)	Pack Absorption
Design 1	2.72–2.79	2.76	97%
Design 2	2.50–2.54	2.52	92%
	5.94–6.14	6.04	84%
Design 3	4.92–5.04	4.98	93%
Final Design	2.47–2.52	2.5	90%
	4.82–4.97	4.9	99%
	5.9–6.11	6	97%

**Table 3:** Permeability and permittivity (real values) at different frequencies.

Mode	Permeability (Less than Zero)	Permittivity (Less than Zero)
TE	4.035–4.95, 4.98–5.96, 6.115–7	2–4.03, 4.925–4.99, 5.955–6.13

**Table 4:** Metamaterial property (real and imaginary values) at the resonant frequency.

EM Mode	Frequency GHz	Permeability		Permittivity		Refractive Index	
		Real	Imaginary	Real	Imaginary	Real	Imaginary
TE	2.5	185.14	−196.032	−83.43	−148.299	26.10	−212.603
	4.9	−58.04	−82.8933	21.67	−89.6942	−17.95	−94.9491
	6	16.27	−49.2774	−52.29	−98.8149	−6.38	−75.90

**Table 5:** Comparisons with existing MMAs.

Ref.	MMA	Size Length × Width × Thickness mm^3^	Substrate	Operating Frequency (GHz)	Absorption %	Metamaterial Property	EMR
[24]	Four C shape ring	40 × 20 × 6.25	PET-PDMS-PET	3.2–11	80%	N/A	2.34
[27]	Split square ring	40 × 40 × 11	PET-PDMS-PET	2.2–5.83	80%	N/A	3.40
[38]	Three square rings	32.4 × 34 × 0.1	PET	1–4.5	90%	-	8.82
[31]	Two modified rings	10 × 10 × 1.6	FR-4	3.36, 3.95, 10.48	92.9%, 96.8%, 99.9%	SNG	8.92
[32]	Three Concentric metallic resonators	10 × 10 × 0.8	FR-4	3.95, 5.92, 9.21	92.2%, 94.5%, 98.7%	N/A	7.59
[33]	Six distinct concentric rings	33.5 × 33.5 × 6	Neoprene rubber	1.75, 2.17, 2.6	96.91%, 96.41%, 90.12%	N/A	5.11
[34]	Triple circular slot ring	14 × 14 × 1	FR-4	2.9, 4.18, 9.25	97%, 96.45%, 98.20%	N/A	7.38
[35]	Circular ring and inner Jerusalem cross	13.8 × 13.8 × 1	FR-4	4.4, 6.05, 13.9	97%	N/A	4.94
[36]	Two C shape square ring	34 × 34 × 3.2	FR-4	2.45 and 5	90%, 99%	N/A	3.60
[37]	Split circular rings	18 × 18 × 1.75	Rogers RO 3003	2.4, 5.1	99%	N/A	6.94
proposed	Square splits ring resonator	9.5 × 9.5 × 1.6	FR-4	2.5, 4.9, 6	90%, 99%, 97%	SNG	15

## Data Availability

The data presented in this study are presented in this article.
